# Preferences for an HIV prevention mobile phone app: a qualitative study among men who have sex with men in China

**DOI:** 10.1186/s12889-019-6617-4

**Published:** 2019-03-12

**Authors:** Aidi Zhang, Nancy R. Reynolds, Jason E. Farley, Xiao Wang, Simin Tan, Jin Yan

**Affiliations:** 1 The Third Xiangya Hospital of Central South University, 138 Tongzipo Road, Changsha, 40013 China; 2 Johns Hopkins University School of Nursing, 525 North Wolfe Street, Baltimore, MD 21205 USA

**Keywords:** Men who have sex with men, HIV prevention, Mobile phone app, Integrated framework

## Abstract

**Background:**

Men who have sex with men (MSM) have a disproportionate burden of HIV infection. Mobile phone apps provide a promising means of improving HIV prevention among MSM. But this has received little examination in China. The objective of this study was to explore MSM’s preferences for an HIV prevention mobile phone app.

**Methods:**

Qualitative semi-structured personal interviews were conducted with 19 MSM to determine their preferences for features and content to inform the design of an app aimed at HIV prevention in China.

**Results:**

Five categories were identified under the main category preferences for features of the app: target population, attributes, language used, potential user access, and perceived usefulness. Five categories were identified under the main category preferences for content of the app: functions to facilitate HIV testing behavior, HIV post-exposure prevention, warning against substance use, psychological support, and areas for communication.

**Conclusions:**

Findings suggest that the design of an app targeting MSM in China should use an integrated framework addressing behavioral and psychological aspects, satisfy common needs of potential users, avoid perpetuating negative stereotypes and stigma, and avoid possible increase of risk behavior due to using the app.

## Background

Men who have sex with men (MSM) have a disproportionate burden of HIV infections. Although MSM account for only 2 to 4% of the Chinese adult male population, MSM accounted for 25.5% of the national annual new cases of Human Immunodeficiency Virus (HIV) infection [1, 2]. High acceptability and wide coverage among MSM are critical characteristics for the HIV preventions to get success [3]. Mobile phone apps have great potential to obtain these features and become a vital window of opportunity to protect MSM from HIV infection and promote their sexual health [4].

The advent of the mobile Internet—especially mobile phone technology has changed the patterns of interaction among MSM populations. Various gay-dating apps have become primary approaches for their social networking. The highly popular app in China, *Blued*, has an estimated 27 million users worldwide. While such apps offer a supportive community and opportunity for social interaction, they may also foster a risk environment for HIV and sexually transmitted infections (STIs) transmission among MSM. Surveys among MSM in China showed that nearly 66% have used gay apps to seek sexual partners in the last six months, 36.4% of gay app users met their last sex partner within 24 h after their first message exchange [5, 6].

At the same time, app also has advantages as tool to deliver risk reduction interventions. First, is the high willingness. Previous studies showed that 63.9–91% of MSM expressed willingness to participate in app-based HIV-prevention programs [7–9]. Second, mobile phones are portable and available to users online 24/7; there is no limitation of time and space. This enables interaction between the phone and the user, between healthcare providers and the receivers more frequent and convenient. Third, app-based interventions have the potential to scale up in a short time and with low costs. It has been suggested that we can only achieve meaningful reduction of HIV infection if prevention services gain a coverage of 40–50% among MSM populations [3]. App-based interventions have great potential to help us accomplish this aim.

Several studies have been conducted with MSM to explore expected content of HIV prevention apps targeting MSM populations. The expected content include: educational modules on HIV prevention and treatment [10, 11]; integrate sex education and safer sex practices into different partner types for younger MSM [12, 13]; HIV testing plans, reminders, and information about testing site locations [8, 14–17]; self -assessment and feedback of behavioral risks and symptoms [9, 14, 16], information and screening for pre-exposure prophylaxis (PrEP) and post-exposure prophylaxis (PEP) [10, 16]; linkage to healthcare providers [16, 17]; communication with other users [14, 17]; commodity (e.g. condoms, lubricants, self-testing kits) ordering [16]. The expected features of mobile apps include: the app must be safe for users, secure their privacy and confidentiality [13]; it should be easy-to-use, interactive, and have content to satisfy the needs of different individuals [10, 14, 15]. Other studies focused on the development of apps to promote adherence of antiretroviral therapy among MSM living with HIV suggest apps should allow management of individual health records and the potential benefit of adding elements such as gamification to increase download and deepen the level of the user’s participation [18–20].

Using an app to promote HIV prevention among MSM in China is still in its infancy. MSM in China have also expressed an interest in receiving HIV prevention services through mobile phones [21]. Since there are diverse cultural differences among MSM and related differences in understanding of HIV risk and help needs for HIV prevention [17], there is a need for studying the preferences of Chinese MSM in order to develop an appropriate HIV prevention aimed app for this population. Further, most of the previous studies exploring functionality of apps for HIV prevention among MSM mainly focused on behavioral changes and paid less attention to psychosocial aspects. The behavioral and psychosocial conditions of MSM are interrelated, the co-occurrence of multiple psychosocial problems, such as depressive conditions and discrimination, could interact to generate additive effects on HIV-related sexual risk behaviors and HIV incidence [22–24]. Thus, HIV prevention interventions should be developed under an integrated framework, combine behavioral with psychosocial aspects. The aim of this study was to identify Chinese MSM’s preferences for features and content of an HIV prevention-aimed app.

## Methods

### Participants and recruitment

Semi-structured personal interviews with MSM were conducted in Changsha, China, about four months from September to December 2015. Inclusion criteria included: (1) biologically and self-reported as male; (2) 18 years of age or older; (3) had same-sex anal intercourse during the last 12 months; (4) owned at least one smart mobile phone; (5) willing and able to give informed consent. Participant exclusion criteria were: (1) unable to participate in the program because of cognitive or psychiatric disorders; (2) had already attended another HIV prevention program.

Purposive sampling and snowball sampling were employed to recruit participants. Participants were found through both online and offline approaches. Advertisements with introduction to the program and contact information were released on two frequently used gay-dating apps: *Blued* and *ZANK*. Participants were also encouraged to recruit other peers into this study.

### Study procedures

Those willing to participate in the interviews were screened for eligibility, consented, and appointments were made with eligible participants to complete the personal interviews. Interviews were conducted by the first author, who is female, in private or quiet places (e.g., the interviewer’s office, quiet zone of a park). Before each interview, the interviewer established rapport with the participant while making a self-introduction, introducing interview aims, procedures and questions, and the participant was promised personal confidentiality and freedom to withdraw from the interview. No other relationship was established between the interviewer and the participant prior to the start of the interview.

All interviews were audio-recorded with the participants’ consent and field notes were made during the interviews. Every participant received an HIV self-testing kit after the interview. Sample size was determined by information saturation [25]. This study was approved by the Institutional Review Board of the Third Xiangya Hospital of Central South University. All identifiable information was removed from study materials (e.g., participant’s name, hometown place, and other information which might help identify the person removed). All study participants were assigned unique study identifiers that appeared on all data collection instruments, tapes, documents, and files.

Individual characteristics were collected before start of the interview, including age, birthplace, sexual orientation, educational background, marital status, occupation, and HIV-testing experience. A semi-structured interview outline was used to facilitate the interviews. A series of open-ended questions were asked regarding participants’ preferred features and content needs of an HIV prevention-aimed app. The questions were revised after the first two interviews, with words and tones of some questions modified, and two questions were added. The main questions were as follows: (1) In your opinion, what are the reasons for the rapidly increasing HIV infection among MSM populations? (2) What factors affect HIV test-taking behavior? (3) If we are going to develop an app aimed at HIV prevention for MSM populations, what are your opinions or preferences about the design of the app (such as name, logo, language)? (4) What contents would you want in such an app? (5) Would you be willing to use the app? Do you have any concerns? (6) What do you think would improve MSM user’s acceptance and long-term use of the app? For the first four questions, we also reminded the participants to consider their responses from a behavioral as well as psychosocial perspective.

### Data analysis

Analysis started concurrently with data collection. Each audio-recorded interview was transcribed within 24 h after the interview. Content analysis was used, and this process includes open coding, creating categories and abstraction [26]. For the open coding, we wrote down as many headings as necessary by reading each interview material twice. The headings were collected to generate categories. After that, each category was classified to belong to a particular group, so all of the categories were grouped under higher order categories. Two researchers independently analyzed the first two interviews and resolved discrepancies by discussion. A coding and categorizing paradigm was built based on the discussions. The first author analyzed the remaining materials. Nvivo 10.0 was used for assisting with data management and analysis.

## Results

### Participant characteristics

Nineteen MSM were recruited and all of them reached eligibility, completed the individual interviews. Interview time ranged from 32 to 86 min. Age-ranges of the participants were from 18 to 31 years, with mean age of 23.3 ± 3.1 (±s) years. Among the participants, three of them had experiences as peer education volunteers, one was living with HIV. All of the participants were unmarried. For sexual orientation, 16 (84.2%) self-reported as homosexuals, and three (15.8%) as bisexuals. One finished high school, the others were in college or had finished college education. For their occupations, 13 (68.4%) were students, including 11 undergraduate students and two postgraduate students, two (10.5%) were freelancers, three (15.8%) were office workers, and one (5%) was a salesman. Sixteen (84.2%) of the participants reported that they had received at least one HIV test before the interview. Although all of the interviews were conducted in Changsha, the participants were from different regions of China, including six provinces: Henan, Heilongjiang, Hunan, Guangxi, Inner Mongolia, and Sichuan.

### Preferences for features of the app

Preferences for features of the app are summarized under five categories as showed in Table 1 and elaborated below.

**Table 1:** Participants’ preferences for features of the app

Category	Description
Targeting population	Specific for the MSM group; Take adolescent MSM into consideration; Design for various populations, with part of the contents specific for MSM group
Attributes of the app	Be professional as well as be interesting; Privacy, not be so apparent for others telling it is about gay or about HIV; Authority, about who design and develop the app, and who provide information of the app; Interactivity, interactions between the user and the app; In accordance with psychosocial characteristics of the MSM group
Language of the app	Tone of language to be friendly; Words used should show respect to MSM group; Easy to understand
Access to the app	Online accesses, putting the app on a gay-dating app; Offline accesses, peers’ recommendation, advertising at gay bars, and cooperating with local Center of Disease Control, organizations for sexual minorities, organizations for HIV prevention at colleges, and merchants friendly to gay groups
Perceived usefulness of the app	Perception of being cared for by the society; Perception of self behavior risk, strengthen self motivation of behavior changing and improve health behavior skills; Reduce communication cost between the user and healthcare providers

### Target population

Participants had different opinions on the target populations for the app. Some preferred that the app be developed specifically for MSM, making it an area free of discrimination and to enhance a sense of belonging.

“*If the app is made for gay and bisexual men, everyone using the app will be free of concerns about whether other users may say bad things about him. We will be relaxed, and feel the app is made for Zi Ji Ren [our own people]*.” (P6, 23 years old, gay, office worker).

Some participants thought that the app should be designed for people of different sexual orientations. They thought this would help it to reach a larger number of potential users, to spread HIV prevention services to various populations, and to enhance mutual understanding between heterosexual and sexual minority populations.

“*Straight people account for the majority of the population, so if the app is only for gay people, it cannot reach a large population and survive in the market. Besides, HIV prevention services should spread to the national population, not just for gays. I think you can make the app for different populations, and have an area about sexual minorities*.” (P8, 24 years old, gay, office worker).

“*What we want most is the acceptance of the society. If the app can be used by gay as well as by straight people, they can have a better understanding of us*.” (P4, 31 years old, gay, office worker).

Some participants also pointed out the importance of taking adolescent sexual minority males into consideration. They thought the app could be a supportive resource at one’s critical period of sexual identity development, building health promotion awareness, motivation and skills.

“*For those adolescent boys who are at a period of realizing their own non-straight sexual orientation, they may have confusion and stresses for same-sex romantic feelings, wondering if there is something wrong with himself. You need to let them know that being a gay is not something abnormal*.” (P12, 20 years old, gay, student).

“*For those boys who newly enter the “Quan Zi” [a name used to stand for gay community by its members], they are pretty vulnerable to temptation, and tend to commit unprotected sex with those who are older and have more experience in the “Quan Zi”*.” (P7, 24 years old, gay, student)

### Attributes of the app

The most frequently mentioned desired attribute was professionality. It was regarded as a prerequisite for reaching the goal of improving user’s health behaviors.

“*If your app is oriented to change MSM’s sexual risk behaviors, knowledge in the app should be professional, like the HIV prevalence among local MSM population, it should be dynamic with the latest updates, and be presented as exact numbers. This can gain trust from the users and be more likely to raise their risk awareness*.” (P1, 23 years old, gay, office worker).

However, participants also pointed out that if the app is too professional, users might get bored. They suggested the app be professional and be interesting simultaneously.

“*The app should be professional as well as be interesting, so it can satisfy different tastes of users, not lead to uninstalling it. You may use different styles for different purposes, such as be professional for the knowledge, and be interesting for news of events*.” (P9, 23 years old, bisexual, student).

Privacy was also a frequently mentioned attribute. Participants suggested the name and logo of the app not be gay-identified or HIV-identified, in case of unintentional disclosure of the user’s sexual orientation, or mistaken by others that the user is HIV infected by seeing the app’s name and logo.

“*There was a guy who told us online that his mom found out he is gay, just last night. His mom found photos of he and other men on his phone, and kept asking him about the photos, and questioned about whether he is gay*.” (P16, 23 years old, gay, student).

“*A password is necessary when entering the app to keep others from opening it when using the user’s phone.*” (P12, 20 years old, gay, student).

“*People will think only HIV infected individuals use the app, and my friend may doubt if I am HIV infected and may keep away from me.*” (P18, 23 years old, gay, student).

Another desired attribute was authority. This can be segmented into two aspects: who designed and developed the app, and who provides the information presented on the app.

“*You need to tell us who developed the app. The users will not have lots of concerns if they know the developer comes from an authoritative institution, such as the Center for Disease Control or a comprehensive hospital with good reputation.*” (P11, 25 years old, gay, freelancer).

“*If the information is provided by experts from HIV/STIs prevention and treatment fields, the users will be more likely to trust it.*” (P14, 22 years old, gay, student).

Interactivity was thought to be important.

“*If you don’t have interactive functionality, like when I finish the behavioral assessments, if I don’t get any feedback or advice, it will be a bad user experience.*” (P9, 23 years old, bisexual, student).

Participants also thought that the contents in the app should be in accordance with psychosocial characteristics of gay groups. This means that the designer and developer should have good knowledge about behaviors and interaction patterns, preferences, and help needs of gay groups.

“*I think your team needs to involve someone very familiar to gay culture. He knows what we need, what we are interested in, and what is going on in our group. And I recommend that you cooperate with an organization such as Dan Lan (developer and operator of the gay-dating app Blued). This can make you understand us better. You know, sometimes straight people may say something inadvertently, but that hurts our dignity.*” (P6, 23 years old, gay, office worker)

### Language of the app

Participants expressed a preference for the tone of language in the app to be friendly.

“*The user won’t feel distance with the app, if it seems like he is talking to another guy, which makes him feel comfortable.*” (P8, 24 years old, gay, office worker).

The words used in the app should show respect to MSM group and avoid potential stigmatization:

“*For example, there is an excellent magazine about feminism in China, every word in its articles is used carefully. Like the phrase “Fu Ru Jie Zhi” (Fu means women, Ru means children, the phrase means the information is known by everyone, even among women and children) may be seen as discrimination of women and children, the journal never uses these kinds of words. Lots of people don’t care about the words during their communication, but some words do make us uncomfortable.*” (P6, 23 years old, gay, office worker).

And the language needs to be easy to understand.

“*Don’t use words too academic to understand. Just tell us the truth we need to know by using words everyone can understand. But not too much funny words, otherwise, people will not take the information seriously.*” (P16, 23 years old, gay, student)

### Accesses to the app

Participants recommended ways of spreading the app to potential users. Putting the app on a gay-dating app popularly used was the way most frequently mentioned.

“*You can reach a large number of users by putting your app into Blued, since it already has more than 27 million users. Besides, your app can be a necessary supplement to the gay dating apps since they lack functions like health promotion and HIV prevention.*” (P10, 31 years old, office worker).

The suggested offline approaches included key figures’ recommendation among MSM groups, advertising at gay bars, disseminating by cooperating with the local Center of Disease Control, organizations for sexual minorities and organizations for HIV prevention at colleges.

Participants also talked about cooperating with merchants friendly to gay groups.

“*You can cooperate with those merchants, having their products such as condoms and lubricants on your app, and people will be willing to use your app if they can get discounts for those products.*” (P4, 31 years old, office worker)

### Perceived usefulness of the app

Participants expressed being cared by the society through the app.

“*The existence of the app will give us the feeling that the gay population is being cared by the society.*” (P3, 22 years old, gay, student).

They thought the app could be a warning for the users, enhance perception of behavior risk for HIV infection, strengthen their motivation of behavior changing, and improve health behavior skills.

“*People always say that HIV is far from oneself, they care little about it. But if you realize your behavior has risk for HIV infection, you will pay more attention.*” (P15, 18 years old, gay, student).

And they thought the app could also be recognized as a way of reducing communication costs with healthcare professionals.

“*An important thing is that without the app, the communication cost with professionals may be very high, including time and transportation costs. The app can help reduce the costs, by including a common knowledge module and individual health counseling service with a health provider.*” (P6, 23 years old, gay, office worker)

### Preferences for content of the app

Preferences for content identified are summarized in Table 2 and elaborated below.

**Table 2:** Participants’ preferences for content of the app

Category	Description
Content to facilitate HIV testing behavior	Educational modules, including the importance and benefits of getting tested, details of testing procedures, window periods of HIV testing, and HIV transmission and treatment. HIV testing resources, a searching function for looking for information about testing site, or notification push about information of nearby testing site; Checking test results through the app; Periodical behavioral risk assessments as self behavior monitoring; Reminders of regularly testing
HIV post-exposure prevention	Including information of under what conditions mean HIV exposure, institutes from where one can get the medicine, medication prescribing procedures, and its side effects
Warning against substance use	Information of harms of substance use and its influence on sexual risk behavior
Psychological support	Education module, including knowledge of human sexual orientation, equity and rights of the sexual minorities, promotion of self-acceptance; Tips and peers’ experience sharing of coming out to parents; Self-assessment of mental health conditions, and advice-offering; Psychological service resources, including resource recommendations, online counseling services, and assistance with appointment making Linkage to resources of building “Xing Hun” (formality marriage)
Areas for communication	Peer communication for problem solutions, for health behavior supervision and encouragement, but avoid making it as a hooking-up area; Communication with multidisciplinary healthcare providers

### Functions to facilitate HIV testing behavior

Participants expressed a need for educational modules to enhance user’s knowledge, including the importance and benefits of getting tested, details of testing procedures, window periods of HIV testing, and HIV transmission and treatment information. They thought this information could be helpful in improving their knowledge about HIV and HIV testing, and clarify misunderstandings. One of the misunderstandings was embarrassing feelings accompanying HIV testing.

“*I feel too embarrassed to get tested at the Center of Disease Control. It is about sex, and what’s more, if the staff asks me about my sexual orientation, it will be more embarrassing.*” (P5, 21 years old, student).

“*I had been a volunteer at HIV testing events. Many people seemed so shy when they came for testing that they left immediately after finishing the testing procedure.*” (P11, 25 years old, gay, freelancer).

Others discussed inaccurate views held by some MSM at risk for HIV.

“*Lots of people fear of test taking. They think AIDS is incurable and will definitely lead to death. They even don’t know people living with HIV can get free medicine for treatment. So they’d rather not go for testing since they hold the view that the infected one will be dead anyway whether he knows infection status or not.*” (P8, 24 years old, gay, office worker).

“*If the person wore a condom every time when having sex, he may think he has good self-protection and has no risk for HIV infection.*” (P1, 23 years old, gay, office worker).

In addition, relationship status may negatively affect HIV testing behavior.

“*If one has a boyfriend, he will think it is important to get tested, be responsible for the other one and for their relationship. But if he has no boyfriend, he may just not care about this. As you know, building a romantic and long-term relationship is so difficult in the “Quan Zi”, I feel disappointed.*” (P19, 24 years old, gay, salesman).

“*I had a boyfriend and I always trusted him. He said he got tested and is negative, so I never went for testing until we broke up. And my testing result turned out to be (positive)..*.” (P5, 21 years old, student).

Participants also expressed the need for information about testing resources, and they suggested two ways resources could be presented at the app. One was a search function for the users to find local testing sites, with information about the addresses, contact approaches, and working hours. In addition, information about attitudes of the working staff toward MSM was discussed as important, should be presented along with other information about the testing site.

“*You could show him a map of testing sites around the country, if he chooses one site, there will be information about the address, transportation methods, and contact numbers, so it is a quick way.*” (P6, 23 years old, gay, office worker).

“*Some of the staff working at the testing site give you the feeling of being despised. And you just don’t want to take another test for as long as possible.*” (P3, 22 years old, gay, student).

Another way suggested was a notification push with information about the testing sites nearby.

“*If I open the app, and there is a notification telling me there is a testing site nearby, there is a great chance I may go get tested.*” (P5, 21 years old, student).

Many participants suggested the function of being able to check test results through the app. The “huge mental stress” of checking test results may make people reluctant to get tested in the future. Participants thought checking testing results through an app is a way to optimize client’s testing experience, as well as improving testing sites’ working efficiency.

“*I went to the local Center of Disease Control for HIV testing, it brought me huge mental stress. I got venous blood for testing, they told me it would take three to seven days before the result is available. I suffered from anxiety every moment during the waiting period. What’s more, I had to call for the result, another huge stress like I’m calling for a trial conclusion.*” (P6, 23 years old, gay, office worker).

Periodical behavioral risk assessments were regarded as a way of self-monitoring of one’s behavior.

“*If the app reminds him to recall sexual behaviors regularly, like every month, and if he had multiple sexual partners, or didn’t use condom, he would realize there is risk and he need to go for testing.*” (P9, 23 years old, bisexual, student).

Participants thought reminders of regularly testing was also needed. Most indicated they would prefer to set testing dates and the reminder push time by themselves on the app.

“*Reminders from the app telling me to get test is necessary. But I’d like to set the reminders myself on the app rather than its automatically setting and pushing, it will be annoying.*” (P6, 23 years old, gay, office worker)

### HIV post-exposure prevention

Six participants talked about HIV post-exposure prevention, or what we called post-exposure prophylaxis (PEP). They suggested that the app should include content addressing what conditions mean HIV exposure, institutes where one can get the medicine, medication prescribing procedures, and its side effects.

On the one hand, participants expressed that they had limited knowledge about PEP. One of the six persons thought PEP involved “getting an injection” instead of taking oral medications.

“*I got the name on the Internet, all I know is one has to take the pills as soon as possible after HIV exposure, but I have no idea how it works, where and how to get those pills.*” (P5, 21 years old, gay, student).

On the other hand, participants thought that providing PEP information, might in some cases, increase risk taking behavior.

“*You must let them know the medicine is not 100% effective, it has side effects. And never take it as an omnipotent HIV prevention shield, it is making fun of your own health if you do so.*” (P2, 22 years old, bisexual, student)

### Warning against substance use

Nine participants pointed out there should be information about the harms of substance use and its influence on sexual risk behavior. Four disclosed they had experience using the substance Rush Poppers, an inhalant used by MSM as recreational drug before or during anal intercourse.

“*You need to put substance use harms on the app. The drug does have an influence on your sexual behavior, you might lose a clear mind and become extremely excited after using it, and be more likely to commit sexual risk behaviors.*” (P16, 23 years old, gay, student).

However, other participants expressed concerns about adding information about substance use in the app, thinking it reinforced stereotypes and stigma toward MSM.

“*Substance using is not so common, I saw someone selling Rush Poppers before, but just a small proportion of the group use it. People only see part of the picture, but they tend to overgeneralize it, and add a label on the whole population.*” (P3, 22 years old, gay, student).

“*The gay population have already been suffering various stigma, not one more.*” (P6, 23 years old, gay, office worker)

### Psychological support

Participants suggested the need for psychological support by expressing tremendous feelings of pressure and confusion about the future, low levels of self-acceptance, the dilemma of whether or not to come-out, lack of faith in building a serious and long-term romantic relationship, lack of hope for having a family structure like heterosexual couples, and, confusion about self-development. Based on the stresses, they suggested resources of different types.

First there was the suggestion of the need for an educational module that includes content about human sexual orientation, equity and rights of sexual minorities, and promotion of self-acceptance. Participants identified experiencing stresses related to a gay identity. One participant expressed a sense of inferiority in comparison to their heterosexual peers, and many of them used the word “Zheng Chang Ren” [normal people] as an alternative of heterosexual populations.

“*Every time I saw a handsome boy with his arm around a girl, I had a feeling of strong inferiority. He is good looking, he has a girlfriend; such a normal way. I have no advantage compared to him.*” (P3, 22 years old, gay, student).

“*This (gay) identity of mine makes me feel like very different from those “Zheng Chang Ren”. Everything I do seems like different, which gives me tremendous stress. I am even afraid of making friends with boys, for fear of being discriminated.*” (P14, 21 years old, gay, student).

Participants also discussed the need for tips and shared experiences of peers’ coming out to parents. They expressed the dilemma of coming out, the desire of being true self and not be forced into marriage with a girl, but fear of disclosure leading to relationship conflicts with parents or bringing social discrimination to them.

“*I struggled for a long time, and I decided to come out to my parents in the future... I don’t want to conceal this for my whole life. And I don’t want to be like other gays, who didn’t come out to their parents, and be urged to marry a woman all the time... But I don’t have a plan yet.*” (P12, 20 years old, gay, student).

“*Although I can get a feeling of relief after coming out to my parents, they will suffer huge stress afterwards. There would be lots of comments and gossips around them. It is not fair for them. Every child should not do this to his parents.*” (P10, 31 years old, office worker).

Second, there was a need for self-assessment of psychological health, and recommendations according to the assessment results.

“*If there are some assessments, such as for depression, the tests taker can gain a general understanding of his own mental health condition. He can also get advice based on the scores, or if it is necessary to get help from professionals.*” (P2, 22 years old, bisexual, student).

Third, was a need for psychological service resources, including resource recommendations, online counseling services, and assistance with appointment making.

“*I do want to get psychological counseling. But the problem is I don’t know where to find the credible resources, especially service specific to sexual minorities. It will be great if the app has function to help the user make an appointment for psychological counseling.*” (P6, 23 years old, gay, office worker).

Three participants talked about the need for help in building heterosexual marriages. They were thinking of getting “Xing Hun” [a pattern of formal marriage or cooperative marriage in which a gay man and a lesbian woman get married]. They expressed the need for resources to find an appropriate girl.

“*I may choose “Xing Hun”. I definitely object to marry a straight girl like the last gay generation did. Those girls were victims, their whole lives were ruined.*” (P19, 24 years old, gay, salesman).

“*It is hard to find an appropriate girl for “Xing Hun”. You need to consider if you two can reach agreements on various things, like whether having a baby, how to cope with parents, financial issues, and so on.*” (P9, 23 years old, bisexual, student).

“*If you can cooperate with websites or other resources serving for “Xing Hun”, it may be a big selling point of your app since there are substantial demands.*” (P4, 31 years old, gay, office worker)

### Areas for communication

Participants expressed the need for opportunity to communicate with peer users as well as healthcare providers through the app.

Participants thought that communication with a peer with similar experience and problems, would allow one to express his own confusions, get a sense of belonging and get tips for solutions from others’ experiences. Peer communication can also be developed as peer education by health promotion, encouragement, and supervision among the users.

“*In reality, we don’t have many friends to talk about things like sexual orientation or HIV, so online communication with other gays is important. It will be better if you can develop an area where we can have communications with each other.*” (P18, 23 years old, gay, student).

“*One can make a group to discuss health topics, and we can get supervision from each other.*” (P1, 23 years old, gay, office worker).

“*You may invite some volunteers or peer educators into the discussion, this will be more acceptable.*” (P17, 24 years old, bisexual, student).

However, participants also thought it might be better to avoid making a peer communication module because it could become a hook-up area.

“*There are more and more people using gay dating apps. The apps are mainly for hooking up, I don’t think there are many benefits. So if you make your app like this, it will become an area for sex, which is the opposite of your goals.*” (P5, 21 years old, gay, student).

An area for communication with multidisciplinary healthcare providers was also suggested. It was thought that the user could use this as an approach to get counsel about behavioral and mental health problems with the providers.

“*Is there any chance to make a quick connection with doctors? Like if I realize there is HIV infection risk or have an emergency problem, I could connect with a health professional quickly.*” (P8, 24 years old, gay, office worker).

## Discussion

In this study, we explored the preferences of Chinese MSM for features and contents of an HIV prevention-aimed app. For features of the app, preferences centered on the targeted populations, attributes of the app, language used in the app, access to the app, and their perception of usefulness of the app. Preferences for content of the app included promotion of health behaviors, such as HIV testing promotion, risk behavior monitoring, and avoidance of substance use, psychological support, such as information of sexuality and development of healthy relationships, self-acceptance, self-assessment for mental health, linkage to psychological counseling services, and even the need for resources for building a heterosexual-type formal marriage, and social support.

Some of the preferences addressed by participants demonstrated the interconnection between behavioral and psychosocial factors. For instance, the content need for HIV test result checking was driven by psychological stress. Another stress driven content preference was information about staff’s attitudes toward MSM. Participants also expressed views about the impact of beliefs and experiences of romantic relationship development on sexual behavior values and practices. Furthermore, although the app is HIV prevention oriented, the existence of the app is perceived by MSM in this study as a sign of being cared for by society.

Some of our findings were similar to findings from other studies, including the features potential users prefer, such as privacy, interactivity, easy to understand and to use, content needs such as educational modules, promotion of HIV testing behavior, connection to healthcare providers, and communication with other users. Instead of mainly focusing on behavioral change, this study highlighted the view that an HIV prevention app for MSM should be designed from an integrated framework addressing behavioral and psychosocial perspectives. Several theoretical frameworks might be employed for the further development of the app. For example, the Information System Research framework [27] could be used to guide the whole processes of app development as iterative cycles including designing, building, and evaluating. The Technology Acceptance Model [28] could be applied for exploring details of perceived usefulness so as to increase MSM’s intention and actual using behaviors of the app. The Information, Motivation, Behavioral Skills model [29, 30] would be helpful for furthering the content development to increase the likelihood of behavioral change.

Participants had different or even opposite opinions regarding some app features, such as being professional or being interesting, the strengths or weaknesses of involving content about PEP and substance usage, and the preference as well as hesitation of needing areas for communication with other users. This suggests that one app cannot satisfy every user’s unique demands, and common issues and needs of the potential users should be emphasized. The findings also indicate that attention should also be given to avoiding potential negative outcomes of the app, such as avoiding having it turn into a hook up app, avoiding possible increase of sexual risk behavior due to using the app, and avoiding user’s perception of being stigmatized from content or language of the app.

There are also topics not mentioned by the participants. Pre-exposure prophylaxis (PrEP) has been regarded as one of the most promising preventions against HIV infection [31]. Involvement of information about PrEP had been talked about in other studies [10, 16], but was not discussed by participants during our interviews. This is likely because of low rate of awareness of, limited access to, and lower interest in taking PrEP in China. PrEP is not covered by health insurance nor widely available in China. Studies show that only 11 to 22% of MSM in China aware of PrEP [32]. Ding et al. found limited desire to use PrEP, among 1033 MSM in China invited to use free daily PrEP, 19.1% expressed willingness to use, but the actual uptake rate was only 2.5% [33]. This also suggests that information about PrEP can be included in an app to enhance knowledge, willingness, and linkage to PrEP.

There are some other interesting findings. One is that adolescent MSM have been put forward as a target population. There has been a serious trend of rapid increase of HIV infections among adolescents and young adults worldwide. In China, during the first half year of 2015, new HIV cases found among populations between 15 and 24 years old increased by 35% compared with condition at the same period in 2014. Among the new cases, 81% got infected by same-sex behavior [34]. Adolescence is a critical period of one’s sexual identity development. Many non-heterosexual identified adolescents tend to make more identity explorations than their heterosexual counterparts. Studies found that sexual minority adolescents often turn to the cyberspace as the main site for testing, clarifying, and performing own sexual orientation identities [35]. However, due to lack of health awareness, and being vulnerable to sexual temptation by those experienced members in the online MSM groups, many of them committed sexual risk behaviors [36]. Therefore, sexual identity educational module should be involved in the app, including sexual orientation, norms and values about sexual and romantic relationships, development of healthy relationships, and safer sex practices.

We also found that many participants are confronting marriage stress. The traditional values of Chinese culture such as “Chuan Zong Jie Dai” [have children to carry on family names], and harmony of “Yin-Yang” [means everything consists of two aspects to reach a harmonious integration, Yin and Yang, when it comes to sex, female stands for Yin and male stands for Yang] makes heterosexual marriage and having children as representation of filial piety and compliance with social expectations. “Xing Hun” is defined as a win-win approach by many MSM, for both the relief of individual’s stress and to fulfill their family’s and society’s expectations. But “Xing Hun” can also bring up various challenges, such as conflicts about financial and child-bearing issues [37, 38]. However, this is a complicated problem related to social norms, family values and marriage values, and may beyond the capacity of an app to assist effectively.

This study has some limitations. One is that we only recruited gay and bisexual identified MSM, as there are also heterosexual identified MSM, their unique preferences may have been missed. Second, since these are qualitative results from a small sample, they may not be generalizable to various MSM groups. Although we tried to include participants with various background, most of the participants in our study were young adults, especially college students. This may make an app designed based on the findings of this study more appropriate to MSM who are college students.

## Conclusions

Despite these limitations, this study is the first of its kind in China to our knowledge, and the findings provide us with a better understanding of the preferences for features and contents of an app tailored for HIV prevention among MSM from the potential users’ perspectives. The findings highlight the importance of having the users in mind across the whole process from designing, developing, to disseminating of the app. An app for HIV prevention among MSM may be more beneficial and acceptable if designed to address both behavioral and psychosocial aspects.
